# The Relationship Between Indicators of Behavior Rhythmicity With Depression, Anxiety, and Stress Among Youths

**DOI:** 10.1155/da/5861864

**Published:** 2026-08-25

**Authors:** Sha Xia, Yue Gong, Mingming Zhang, Zehui Fan, Jian Zhang, Jixin Zhang, Shengnan Li, Xiaojie Dong, Ling-jie Liu, Yan Li, Xiu-qin Hong, Yi-de Yang

**Affiliations:** ^1^ Key Laboratory of Molecular Epidemiology of Hunan Province, Hunan Normal University, Changsha 410081, China, hunnu.edu.cn; ^2^ School of Public Health, Hunan Normal University, Changsha 410081, China, hunnu.edu.cn; ^3^ Hunan Provincial People’s Hospital Affiliated to Hunan Normal University, Changsha 410000, Hunan, China

**Keywords:** anxiety, chronotype, college students, depression, eating jet lag, social jet lag, stress

## Abstract

**Background:**

Chronotype, social jetlag (SJL), eating jetlag (EJL), eating windows, and breakfast skipping are important and commonly used indicators for behavior rhythmicity. The present study comprehensively examined the behavior rhythmicity and depression, anxiety, and stress among university students.

**Methods:**

A cross‐sectional study of a total of 734 college students was conducted. The questionnaire included investigation of sociodemographic characteristics and lifestyle information. Behavior rhythmicity was measured by the Munich Chronotype Questionnaire (MCTQ), the Reduced Morningness–Eveningness Questionnaire (rMEQ), and the Chrononutrition Profile‐Questionnaire (CP‐Q). Depression, anxiety, and stress were measured by the Depression, Anxiety, and Stress Scale‐21 Items (DASS‐21).

**Results:**

A total of 734 participants with completed data were included in this study. After adjustment of age, sex, exercise time, screen time, being the only child, and family’s monthly per capita income, higher sleep‐corrected SJL (SJLsc) was significantly associated with increased risks of depression (OR = 1.527 per 1‐h increase; 95% CI: 1.129–2.066), anxiety (OR = 1.324 per 1‐h increase; 95% CI: 1.029–1.703), and stress (OR = 1.524 per 1‐h increase; 95% CI: 1.112–2.088). Compared with the non‐evening type group, evening chronotype participants had a significantly higher risk of depression (OR = 1.856, 95% CI: 1.228–2.803), anxiety (OR = 1.598, 95% CI: 1.159–2.202), and stress (OR = 1.945, 95% CI: 1.261–2.999). Further stratified analysis by sex showed that the associations were sex‐specific. SJL_sc_ was associated with depression only in males (OR = 2.014, 95% CI: 1.213–3.345), but not in females. Evening chronotype was related to a higher risk of depression (OR = 1.853, 95% CI: 1.130–3.037) and stress (OR = 2.031, 95% CI: 1.227–3.363) only in females. The last eating occasion jet lag was associated with stress in males (OR = 1.383, 95% CI: 1.035–1.846), but not in females. Eating window jet lag was associated with stress in females (OR = 1.155, 95% CI: 1.015–1.315) but not in males.

**Conclusion:**

SJLsc and evening chronotype are significantly associated with higher risk of depression, anxiety, and stress. Sex‐specific associations between last eating occasion jet lag, eating window jet lag, and chronotype with depression, anxiety, and stress existed in young adults.

## 1. Introduction

Mental health problems, such as depression, anxiety, and stress, are rising worldwide, especially in college students nowadays [1, 2]. A study has shown that the prevalence of subthreshold depression among Chinese college students is ~39.7% [3]. Mental health symptoms among Chinese college students are closely related to abnormalities in the circadian rhythm and dietary rhythm [4, 5]. Current research suggests a potential correlation between circadian rhythmicity of behavior (including sleep, social jetlag [SJL], or diet) and depression, anxiety, and stress [6, 7]. However, no previous study has comprehensively investigated behavior rhythmicity, including chronotype, SJL, and diet rhythmicity among young adults, and their potential association with depression, anxiety, and stress.

Circadian rhythm is controlled by a master biological clock located in the suprachiasmatic nucleus of the hypothalamus, regulating sleep, dietary intake, and wakefulness‐sleepiness patterns [8]. In order to better categorize individuals based on their preferred bedtime, we obtain a continuous spectrum of what are known as “chronotypes” primarily divided into morning types, intermediate types, and evening types [9]. Studies have shown that normal work schedules often cause individuals with evening chronotypes to accumulate a substantial sleep debt during weekdays, which they can only compensate for by extending their sleep duration on weekends. This results in SJL, a mismatch between the endogenous biological clock and the external social clock [10]. Long‐term exposure to significant SJL may have adverse effects on one’s health, such as depression [11], obesity [12], and cardiovascular diseases [13].

Dietary rhythmicity, also known as chrononutrition, represents a newly emerging and popular research field that integrates dietary patterns (including meal timing, eating window, and eating jetlag [EJL]) with the body’s natural rhythms [14]. It is a discipline dedicated to studying the impact of the timing and rhythmicity of food intake. Previous studies have found that irregular dietary rhythmicity can easily lead to metabolic disturbances, potentially influencing the risk of obesity [15] and cardiovascular diseases [16]. However, there has been limited exploration of its relationship with depression, anxiety, and stress [17], and no research has yet investigated the correlation of dietary rhythmicity in college students. EJL, eating windows, and weekly breakfast skipping are all core indicators for dietary rhythmicity [18].

Previous studies on the relationship between circadian rhythm and depression, anxiety, and stress are mainly focused on adolescents and shift workers, but the current research in this area is relatively limited [19–21]. Many studies have suggested a correlation between SJL and a higher risk of anxiety or depression [22–24], while at the same time, several studies have concluded that no significant relationship was found [21, 25]. Meanwhile, research on the relationship between dietary rhythmicity and depression, anxiety, and stress is rare; only one study was conducted in a specific population (airline workers) [17], and no previous study has been conducted in young adults.

In the present study, we investigated the prevalence of depression, anxiety, and stress among college students in Hunan, China. Additionally, we explored the correlation between depression, anxiety, and stress and behavior rhythmicity indicators (SJL, sleep‐corrected SJL [SJLsc], chronotype, and sleep duration; and dietary rhythmicity including eating windows, first eating occasion jet lag, last eating occasion jet lag, eating window jet lag, and breakfast skipping).

## 2. Materials and Methods

### 2.1. Participants

The required sample size was calculated to be 187 (for depression), 153 (for anxiety), and 410 (for stress), assuming a significance level of 5%, and an expected prevalence of 33.98% (depression), 38.53% (anxiety), and 18.98% (stress) [26]. In total, 734 individuals participated in the current study, which exceeded the calculated minimum required sample size. The present cross‐sectional study recruited Chinese college students (*N* = 734) from a university in Hunan, China, aged 18–22 years old, from September 2023 to June 2024. Participants meeting the following criteria were included in the study: (1) participants who provided signed informed consent form and (2) individuals who have completed the survey and answered the questions conscientiously.

### 2.2. Procedure

This study was approved by the Ethics Committee of Hunan Normal University (Approval Number 2023‐254), and the research process strictly adhered to the ethical guidelines outlined in the Declaration of Helsinki as amended in 1989. After obtaining informed consent, participants completed a questionnaire survey. The questionnaire content included sociodemographic characteristics, lifestyle information, the Depression, Anxiety, and Stress Scale‐21 Items (DASS‐21), the Munich Chronotype Questionnaire (MCTQ), the Reduced Morningness–Eveningness Questionnaire (rMEQ), and the Chrononutrition Profile‐Questionnaire (CP‐Q). The questionnaire information was obtained through face‐to‐face interviews. Data on a total of 805 participants were collected. After excluding samples that did not meet the requirements or had irregular answers, 734 samples were included in the final analysis.

### 2.3. Measures

#### 2.3.1. Psychological States

The DASS‐21 was used to measure the status of depression, anxiety, and stress among college students [27]. The DASS‐21 comprises three subscales, including depression, anxiety, and stress, with each subscale containing seven items. Each item was answered with a 4‐point scale (0 = did not apply to me at all; 3 = applied to me most of the time). The total score of each subscale was multiplied by two to produce a final score of 0–42. The criteria for dividing the three subscales are shown in Table S1. Next, we grouped the levels of severity into normal–mild and moderate–extremely severe for each score. Normal to mild = 0, moderate to extremely = 1 [28].

#### 2.3.2. Chronotype

The rMEQ assesses students’ chronotypes and consists of 5 items with a total score of 25. Scores are categorized as follows: 22–25 indicate definitely morning type, 18–21 moderately morning type, 12–17 neither type, 8–11 moderately evening type, and 4–7 definitely evening type. For analytical purposes, we further classified scores into two broader categories: ≤11 as the evening type and ≥12 as the non‐evening type (with subcategories of 12–17 for the intermediate type and ≥18 for the morning type) [29].

SJL measurement was performed using the MCTQ. SJL was calculated as the absolute difference between the sleep midpoint on free days and workdays (|MSF‐MSW|) (1 h). In order to eliminate the influence of sleep debt, which refers to compensating for the lack of sleep time during weekdays on weekends, SJL_sc_ was calculated as sleep onset on free days and workdays (SJL_sc_ = |MSF_SC_‐MSW_SC_| = SO_f_ + 1/2 SD_week_‐(SO_w_ + 1/2 SD_week_) = SO_f_−SO_w_) (1 h) [30]. Figure S1 displays the formulas used to calculate SJL and SJLsc. Sleep duration was obtained by the mean sleep duration on working days and two free days per week, as standard [31].

#### 2.3.3. Dietary Rhythmicity

Dietary rhythmicity, including EJL measurement, was performed using the CP‐Q with 18 items [32], the details of this questionnaire are described elsewhere [33]. The eating window was calculated as the interval between the first eating occasion and the last eating occasion in a metabolic day (eating window = time of last eating occasion − time of first eating occasion). Additionally, breakfast skipping was categorized as no (0 = 7 days/week) and yes (1 = <7 days/week). The first eating occasion jet lag was calculated as the difference in first eating occasion timing between weekends and weekdays (|time of first eating occasion on weekends − time of first eating occasion on weekdays|). The last eating occasion jet lag was calculated as the difference in last eating occasion timing between weekends and weekdays (|time of last eating occasion on weekends − time of last eating occasion on weekdays|). The eating window jet lag was calculated as the difference in eating window between weekdays and weekends (|eating window on weekends − eating window on weekdays|) [34].

#### 2.3.4. Covariates

The characteristics of sociodemographics were collected by a self‐designed demographic questionnaire, including age, gender (1 = male, 2 = female), being an only child (0 = yes, 1 = no), family’s monthly per capita income (1 = <3000 RMB/month, 2 = ≥3000 RMB/month, 3 = unclear), exercise time (0 = >1 h/day, 1 = ≤1 h/day), and screen time (0 = ≤7 h/day, 1 = >7 h/day).

### 2.4. Statistical Analyses

The study employed IBM SPSS version 26.0 (version 9.05; SPSS Inc., Chicago, IL) and R 4.2.2 for data analysis. Continuous variables were described using mean and standard deviation (M ± SD), while categorical variables were summarized by prevalence or proportion (*N* [%]). Differences in variables between different chronotype groups were compared with the *T*‐test (quantitative variables with normal distribution) or chi‐square test (categorical variables). Furthermore, to examine associations between behavior rhythmicity (including dietary rhythmicity variables, sleep duration, chronotype, SJL and SJLsc) and the risk of depression, anxiety, and stress, multivariable logistic regression analysis was performed. Model 1 was the crude model. In Model 2, gender and age were adjusted. In Model 3, gender, age, exercise time, screen time, being an only child, and family’s monthly per capita income were all adjusted. Additionally, subgroup analysis by gender was conducted, and interaction was explored by including the interaction item in the logistic models. To verify the robustness of the findings, sensitivity analysis stratified by two distinct outcome definitions combining clinical diagnostic data and DASS‐21 scale scores of mental disease was also conducted. Since clinical data were only available for medical students, sensitivity analysis was limited to a sample of 505 medical students. For Outcome 1, participants with a positive clinical diagnosis or severe/extremely severe DASS‐21 symptoms were defined as the case group; for Outcome 2, individuals with a positive clinical diagnosis or moderate, severe, or extremely severe DASS‐21 symptoms were categorized as cases. In both outcome definitions, controls consisted of participants with negative clinical diagnoses and DASS‐21 scores indicating normal to mild symptom severity. Individuals who had negative clinical diagnoses but moderate, severe, or extremely severe DASS‐21 screening results were excluded. The *p* value <0.05 was considered significant.

## 3. Results

### 3.1. Characteristics of the Study Population

This study included 734 Chinese college students (mean age: 19.08 ± 1.20 years; 27.2% male). The general characteristics of the study population are shown in Table 1. Key measures (mean ± SD) were as follows: eating window (10.97 ± 2.13 h), sleep duration (7.45 ± 0.68 h), SJL (0.79 ± 0.54 h), SJLsc (0.61 ± 0.60 h), first/last eating occasion jet lag (2.33 ± 1.70 h; 0.74 ± 1.15 h), and eating window jet lag (2.24 ± 1.80 h). Additionally, 16.5% (*n* = 121), 37.9% (*n* = 278), and 14.0% (*n* = 103) reported moderate‐to‐extremely severe depression, anxiety, and stress, respectively.

**Table 1:** General characteristics of the study population.

Variable	Total	Non‐evening type	Evening type	*p*
Age	19.08 ± 1.20	19.03 ± 1.24	19.18 ± 1.13	0.133
Gender				
Male	200 (27.2%)	151 (30.8%)	49 (20.1%)	**0.002**
Female	534 (72.8%)	339 (69.2%)	195 (79.9%)	—
Eating window (h)	10.97 ± 2.13	11.03 ± 2.10	10.86 ± 2.21	0.296
Sleep duration (h)	7.45 ± 0.68	7.51 ± 0.66	7.34 ± 0.71	**<0.001**
Social jetlag (h)	0.79 ± 0.54	0.70 ± 0.50	0.97 ± 0.58	**<0.001**
Sleep‐corrected social jetlag (h)	0.61 ± 0.60	0.53 ± 0.52	0.78 ± 0.70	**<0.001**
First eating occasion jet lag (h)	2.33 ± 1.70	2.02 ± 1.53	2.94 ± 1.87	**<0.001**
Last eating occasion jet lag (h)	0.74 ± 1.15	0.72 ± 1.12	0.78 ± 1.20	0.490
Eating window jet lag (h)	2.34 ± 1.80	2.10 ± 1.71	2.81 ± 1.90	**<0.001**
Depression				
Normal‐mild	613 (83.5%)	422 (86.1%)	191 (78.3%)	**0.007**
Moderate‐extremely severe	121 (16.5%)	68 (13.9%)	53 (21.7%)	—
Anxiety				
Normal‐mild	456 (62.1%)	322 (65.7%)	134 (54.9%)	**0.005**
Moderate‐extremely severe	278 (37.9%)	168 (34.3%)	110 (45.1%)	—
Stress				
Normal‐mild	631 (86.0%)	435 (88.8%)	196 (80.3%)	**0.002**
Moderate‐extremely severe	103 (14.0%)	55 (11.2%)	48 (19.7%)	—
Family’s monthly per capita income				
<3000 RMB/month	163 (22.2%)	110 (22.4%)	53 (21.7%)	0.419
≥3000 RMB/month	368 (50.1%)	235 (48.0%)	133 (54.5%)	—
Unclear	203 (27.7%)	145 (29.6%)	58 (23.8%)	—
Being an only child				
Yes	182 (24.8%)	116 (23.7%)	66 (27.0%)	0.318
No	552 (75.2%)	374 (76.3%)	178 (73.0%)	—
Exercise time				
>1 h/day	81 (11.0%)	53 (10.8%)	28 (11.5%)	0.788
≤1 h/day	653 (89.0%)	437 (89.2%)	216 (88.5%)	—
Screen time				
≤7 h/day	269 (36.6%)	197 (40.2%)	72 (29.5%)	**0.005**
>7 h/day	465 (63.4%)	293 (59.8%)	172 (70.5%)	—
Sleep duration, *n* (%)				
≥7 h/day	551 (75.1%)	389 (79.4%)	162 (66.4%)	**<0.001**
<7 h/day	183 (24.9%)	101 (20.6%)	53 (21.7%)	—
Breakfast skipping				
7 days/week	249 (33.9%)	196 (40.0%)	191 (78.3%)	**<0.001**
<7 days/week	485 (66.1%)	294 (60.0%)	164 (67.2%)	—
Social jetlag, *n* (%)				
<2 h	716 (97.5%)	483 (98.6%)	233 (95.4%)	**<0.001**
≥2 h	18 (2.5%)	7 (1.4%)	11 (4.5%)	—
Sleep‐corrected social jetlag, *n* (%)				
<2 h	702 (70.8%)	475 (96.9%)	227 (93.0%)	**<0.001**
≥2 h	32 (4.4%)	15 (3.1%)	17 (7.0%)	—
First eating occasion jet lag, *n* (%)				
<2 h	333 (45.4%)	258 (25.7%)	75 (30.3%)	**<0.001**
≥2 h	401 (54.6%)	232 (47.3%)	169 (69.3%)	—
Last eating occasion jet lag, *n* (%)				
<2 h	647 (88.1%)	430 (87.8%)	165 (88.9%)	**<0.001**
≥2 h	87 (11.9%)	60 (12.2%)	27 (11.1%)	—
Eating window jet lag, *n* (%)				
<2 h	334 (45.5%)	251 (51.2%)	122 (34.0%)	**<0.001**
≥2 h	400 (54.5%)	239 (48.8%)	161 (66.0%)	—

Note: Bold *p*‐values indicate statistically significant associations at the threshold of *p* < 0.05.

### 3.2. Association Between Behavior Rhythmicity With Depression, Anxiety, and Stress

The association between behavior rhythmicity (including dietary rhythmicity variables, sleep duration, chronotype, SJL and SJLsc) with depression, anxiety, and stress are shown in Table 2. Dietary rhythmicity variables include eating windows, first eating occasion jet lag, last eating occasion jet lag, eating window jet lag, and breakfast skipping.

**Table 2:** Associations between behavior rhythmicity indicators with depression, anxiety, and stress.

Dependent variables	Independent variables	Model 1			Model 2				Model 3		
		OR (95% CI)	*p*		OR (95% CI)		*p*		OR (95% CI)		*p*
Depression	Eating window	0.951 (0.868–1.042)	0.283		0.943 (0.860–1.033)		0.205		0.943 (0.860–1.034)		0.213
	Social jetlag	1.347 (0.956–1.897)	0.089		1.400 (0.993–1.974)		0.055		1.385 (0.983–1.952)		0.063
	Sleep‐corrected social jetlag	1.532 (1.142–2.054)	**0.004**		1.531 (1.137–2.062)		**0.005**		1.527 (1.129–2.066)		**0.006**
	First eating occasion jet lag	1.080 (0.965–1.208)	0.182		1.105 (0.986–1.238)		0.086		1.101 (0.983–1.234)		0.097
	Last eating occasion jet lag	1.096 (0.936–1.283)	0.255		1.084 (0.925–1.271)		0.317		1.090 (0.929–1.280)		0.289
	Eating window jet lag	1.025 (0.921–1.141)	0.65		1.036 (0.931–1.154)		0.513		1.042 (0.936–1.161)		0.454
	Sleep duration										
	>7 h	1 (Ref.)	—	1 (Ref.)			—		1 (Ref.)		—
	≤7 h	1.409 (0.917–2.164)	0.117		1.393 (0.905–2.146)		0.132		1.361 (0.881–2.103)		0.165
	Chronotype										
	Non‐evening type	1 (Ref.)	—	1 (Ref.)			—		1 (Ref.)		—
	Evening type	1.722 (1.157–2.563)	**0.007**		1.893 (1.260–2.844)		**0.002**		1.856 (1.228–2.803)		**0.003**
	Chronotype										
	Definitely morning type	NA	—	NA			—		NA		E
	Moderately morning type	1 (Ref.)	—	1 (Ref.)			—		1 (Ref.)		—
	Neither type	0.827 (0.306–2.234)	0.708	0.822 (0.302–2.236)		0.702		0.782 (0.284–2.154)		0.635	
	Moderately evening type	1.503 (0.549–4.116)	0.428	1.628 (0.590–4.497)		0.347		1.524 (0.544–4.270)		0.423	
	Definitely evening type	0.867 (0.184–4.093)	0.857	1.031 (0.216–4.923)		0.97		0.975 (0.198–4.624)		0.975	
	Breakfast skipping										
	7 day/week	1 (Ref.)	—	1 (Ref.)			—		1 (Ref.)		—
	<7 day/week	1.524 (0.985–2.358)	0.059		1.551 (0.999–2.407)		**0.05**		1.473 (0.946–2.292)		0.087
Anxiety	Eating window	0.999 (0.932–1.072)	0.981		0.995 (0.928–1.068)		0.891		0.995 (0.927–1.068)		0.895
	Social jetlag	0.996 (0.758–1.311)	0.98		0.990 (0.751–1.304)		0.942		0.980 (0.743–1.292)		0.884
	Sleep‐corrected social jetlag	1.331 (1.037–1.708)	**0.025**		1.336 (1.041–1.715)		**0.023**		1.324 (1.029–1.703)		**0.029**
	First eating occasion jet lag	1.073 (0.983–1.170)	0.116		1.076 (0.985–1.175)		0.104		1.072 (0.981–1.171)		0.125
	Last eating occasion jet lag	1.125 (0.990–1.278)	0.071		1.119 (0.985–1.272)		0.085		1.122 (0.986–1.279)		0.080
	Eating window jet lag	1.070 (0.985–1.161)	0.109		1.069 (0.984–1.161)		0.113		1.070 (0.985–1.163)		0.110
	Sleep duration										
	>7 h	1 (Ref.)	—	1 (Ref.)			—		1 (Ref.)		—
	≤7 h	1.304 (0.927–1.833)	0.127		1.290 (0.917–1.815)		0.144		1.271 (0.902–1.793)		0.171
	Chronotype										
	Non‐evening type	1 (Ref.)	—	1 (Ref.)			—		1 (Ref.)		—
	Evening type	1.573 (1.150–2.153)	**0.005**		1.610 (1.172–2.211)		**0.003**		1.598 (1.159–2.202)		**0.004**
	Chronotype										
	Definitely morning type	NA	—	NA			—		NA		—
	Moderately morning type	1 (Ref.)	—	1 (Ref.)			—		1 (Ref.)		—
	Neither type	1.852 (0.781–4.393)	0.162	1.831 (0.771–4.348)		0.170		1.733 (0.725–4.142)		0.216	
	Moderately evening type	2.838 (1.175–6.859)	0.020	2.865 (1.183–6.936)		0.020		2.708 (1.111–6.605)		**0.028**	
	Definitely evening type	2.571 (0.769–8.594)	0.125	2.673 (0.796–8.973)		0.112		2.444 (0.721–8.281)		0.151	
	Breakfast skipping										
	7 day/week	1 (Ref.)	—	1 (Ref.)			—		1 (Ref.)		—
	<7 day/week	0.957 (0.699–1.311)	0.786		0.971 (0.708–1.331)		0.853		0.936 (0.680–1.287)		0.682
Stress	Eating window	1.028 (0.932–1.134)	0.579		1.027 (0.931–1.133)		0.589		1.026 (0.929–1.132)		0.608
	Social jetlag	0.914 (0.619–1.349)	0.65		0.927 (0.628–1.369)		0.703		0.920 (0.621–1.361)		0.675
	Sleep‐corrected social jetlag	1.539 (1.134–2.088)	**0.006**		1.532 (1.128–2.082)		**0.006**		1.524 (1.112–2.088)		**0.009**
	First eating occasion jet lag	1.044 (0.925–1.178)	0.485		1.050 (0.930–1.186)		0.431		1.049 (0.928–1.185)		0.445
	Last eating occasion jet lag	1.203 (1.028–1.407)	**0.021**		1.203 (1.028–1.407)		**0.021**		1.213 (1.034–1.422)		**0.017**
	Eating window jet lag	1.077 (0.964–1.204)	0.191		1.081 (0.967–1.209)		0.17		1.092 (0.976–1.222)		0.123
	Sleep duration										
	>7 h	1 (Ref.)	—	1 (Ref.)			—		1 (Ref.)		—
	≤7 h	1.214 (0.762–1.935)	0.415		1.216 (0.762–1.940)		0.411		1.166 (0.728–1.867)		0.524
	Chronotype										
	Non‐evening type	1 (Ref.)	—	1 (Ref.)			—		1 (Ref.)		—
	Evening type	1.937 (1.270–2.955)	**0.002**		1.991 (1.299–3.053)		**0.002**		1.945 (1.261–2.999)		**0.003**
	Chronotype										
	Definitely morning type	NA	—	NA		—			NA	—	
	Moderately morning type	1	—	1		—			1	—	
	Neither type	0.498 (0.195–1.273)	0.146	0.500 (0.195–1.281)		0.149		0.453 (0.174–1.180)		0.105	
	Moderately evening type	0.939 (0.362–2.435)	0.896	0.969 (0.373–2.522)		0.949		0.860 (0.325–2.274)		0.760	
	Definitely evening type	2.083 (0.584–7.431)	0.258	2.225 (0.619–7.996)		0.220		2.098 (0.574–7.672)		0.263	
	Breakfast skipping										
	7 days/week	1 (Ref.)	—	1 (Ref.)			—		1 (Ref.)		—
	<7 days/week	1.049 (0.674–1.633)	0.833		1.046 (0.671–1.629)		0.844		0.984 (0.628–1.541)		0.944

*Note:* Model 1: Crude model. Model 2: Gender and age were adjusted. Model 3: Gender, age, exercise time, screen time, being an only child, and family’s monthly per capita income were all adjusted. Bold *p*‐values indicate statistically significant associations at the threshold of *p* < 0.05.

Abbreviation: NA, not available.

After adjustment for gender and age, higher SJL (SJLsc) was significantly associated with increased risks of depression (OR = 1.531 per 1‐h increase; 95% CI: 1.137–2.062), anxiety (OR = 1.336 per 1‐h increase; 95% CI: 1.041–1.715), and stress (OR = 1.532 per 1‐h increase; 95% CI: 1.128–2.082). Breakfast skipping was independently associated with depression, while greater last eating occasion jet lag was specifically linked to higher stress risk.

After adjustment for gender and age, breakfast skippers showed higher odds of depression compared to daily breakfast consumers (OR = 1.551; 95% CI: 0.999–2.407). Each 1‐unit increase in last eating occasion jet lag was associated with increased stress risk (OR = 1.203; 95% CI: 1.028–1.407). After adjustment for gender, age, exercise time, screen time, being an only child, and family’s monthly per capita income, evening‐type participants had significantly higher odds of depression (OR = 1.856; 95% CI: 1.228–2.803), anxiety (OR = 1.598; 95% CI: 1.159–2.202), and stress (OR = 1.945; 95% CI: 1.261–2.999) than non‐evening types.

We also analyzed the logistic regression analysis of the dietary rhythmicity variables and chronotype with depression, anxiety, and stress in the two categories and three categories. Generally similar results were obtained; the results are shown in Table S2.

Additionally, we performed linear regression analyses on the depression, anxiety, and stress scores from the DASS‐21 scale, as well as their total scores, with the dietary rhythmicity variables and chronotype; the results are shown in Table S3.

### 3.3. Stratified Analysis by Sex of the Association Between Behavior Rhythmicity With Depression, Anxiety, and Stress

**Figure 1:**
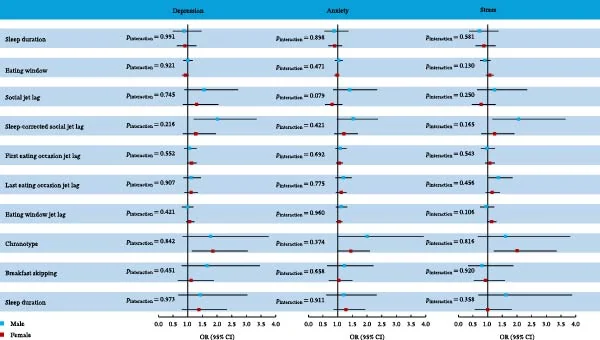
Interactive effects between gender and behavior rhythmicity indicators on depression, anxiety, and stress. Depression, anxiety, and stress were categorized as binary variables (normal‐to‐mild vs. moderate‐to‐extreme). Rhythmicity indicators including sleep duration, eating window, social jetlag, sleep‐corrected social jetlag, first eating occasion jet lag, last eating occasion jet lag, and eating window jet lag were analyzed as continuous variables. Rhythmicity indicators, including chronotype and breakfast skipping, were analyzed as categorical variables: chronotype (non‐evening [reference] vs. evening), breakfast skipping (no vs. yes), and sleep duration (≥7 vs. <7 h). Gender, age, exercise time, screen time, being an only child, and family’s monthly per capita income were all adjusted.

Stratified analysis by sex of the association between behavior rhythmicity with depression, anxiety, and stress is shown in Figure 1. Detailed data are presented in Table S4. After adjustment of sex, age, exercise time, screen time, being an only child, and family’s monthly per capita income, we found that SJL_sc_ is associated with risk of depression in males (OR = 2.014, 95% CI: 1.213–3.345); however, it is not significant in females. In females, there was a correlation between chronotype and both risk of depression (OR = 1.853, 95% CI: 1.130–3.037) and stress (OR = 2.031, 95% CI: 1.227–3.363), whereas no significant correlation was found in males. Additionally, in males, there was a significant correlation between the last eating occasion jet lag and stress (OR = 1.383, 95% CI: 1.035–1.846), but not in females. In females, there was a correlation between the eating window jet lag and stress (OR = 1.155, 95% CI: 1.015–1.315), but not in males. Furthermore, there was a correlation between chronotype and anxiety in both males (OR = 2.013, 95% CI: 1.031–3.929) and females (OR = 1.453, 95% CI: 1.009–2.093).

However, when the interaction item between behavior rhythmicity profile variables and sex was included in the logistic regression analysis, no significant interaction effect was found on depression, anxiety, and stress.

For the sensitivity analysis restricted to 505 medical students with complete clinical diagnostic data and DASS‐21 scale data, defined by the two separate outcome criteria above, logistic regression models were constructed separately for Outcomes 1 and 2. The findings derived from both sensitivity outcome definitions were generally consistent with the primary analysis, thereby corroborating the robustness of our core research conclusions. Detailed results of this sensitivity analysis are presented in Table S5.

## 4. Discussion

The present study found that SJLsc and evening chronotype are associated with depression, anxiety, and stress. In addition, sex‐specific associations between last eating occasion jet lag, eating window jet lag, and chronotype with depression, anxiety, and stress existed in young adults. As far as we know, this is the first study comprehensively investigating the behavior rhythmicity profile with depression, anxiety, and stress among young adults.

We found that an increase in SJL_sc_ is significantly correlated with the risk of depression, anxiety, and stress, which is consistent with the findings of Li et al. [24] in a study of adolescents. A consistent link between circadian misalignment and stress was also reported in Socci et al.’s [35] study. Qu et al. [36] also found that SJL was statistically positively associated with moderate and above depressive symptoms after conducting a study on college students in Anhui and Jiangxi provinces in China. We found that SJL_sc_ is associated with risk of depression in males (OR = 2.014, 95% CI: 1.213–3.345); however, it is not significant in females. No significant association between SJL and the first eating occasion jet lag with depression, anxiety, and stress problems was found. After adjusting for sex and age, there is a significant correlation between breakfast skipping and the risk of anxiety, consistent with the research findings of Qiao et al. [37]. There are two possible mechanisms for the association between dietary rhythmicity and depression, anxiety, and stress: first, irregular mealtimes are significantly associated with metabolic alterations, including dysregulation of leptin and growth hormone‐releasing peptide functions, which may subsequently contribute to the impairment of depression, anxiety, and stress; second, dietary jet lag may alter the methylation of five crucial genes and may be associated with a range of metabolic health issues; it may also be a factor in impaired depression, anxiety, and stress by reducing neuronal complexity, cognitive flexibility, and executive function [38, 39].

An increase in the last eating occasion jet lag is significantly associated with the risk of stress. Our study found that individuals with eating window jet lag ≥2 h were more prone to depression compared to those with eating window jet lag of <1 h. Additionally, individuals with eating window jet lag of ≥1 h but <2 h were more likely to experience stress compared to those with eating window jet lag of <1 h. Similar to a cross‐sectional study from 2022 to 2024 conducted by Zhang et al. [17], they also found that long eating windows were associated with an increased risk of depression and anxiety among airline workers. In males, there was a significant correlation between the last eating occasion, jet lag, and stress, but not in females. In females, there was a correlation between the eating window, jet lag, and stress, but not in males. Compared with the non‐evening type, evening‐type participants had a higher risk of depression, anxiety, and stress, which is consistent with the findings of Magnusdottir, Tonon, and Evans et al. studies [40–42]. Specifically, Riccobono et al.’s [43] study on Italian college students found a correlation between higher BDI scores and lower MEQ scores, which aligns with our finding that evening‐type participants had an elevated risk of depression. In females, there was a correlation between chronotype and both risk of depression and stress, whereas no significant correlation was found in males. There was a correlation between chronotype and anxiety in both males and females. Jung and Lee [44] found that short sleep duration (<6 h), evening chronotype, and SJL (>2 h) were significantly associated with anxiety disorders in women, while only short sleep duration (<6 h) was linked to anxiety in men. These sex differences warrant further investigation.

There are limitations in the present study. First, this study has several limitations. First, the cross‐sectional design prevents us from establishing causal relationships between circadian rhythm variations and depression, anxiety, or stress among college students. Evanger et al.’s [45] longitudinal research revealed that baseline morningness predicted lower anxiety and depression risk at follow‐up, while evening preference was associated with a higher depression risk than intermediate preference. These findings highlight the need for future longitudinal studies to clarify these complex associations. Another limitation is that the study did not assess drug use or collect information regarding participants’ potential mental disorders or medication status—factors that could significantly influence all examined variables. Furthermore, given that all data were collected via self‐reported questionnaires, the subjectivity of the data inevitably introduces bias. Additionally, the sample comes from a single university and is predominantly female, which restricts generalizability. Therefore, future studies should consider incorporating objective measures to validate and augment these findings. Finally, the DASS‐21 scale is merely a simplified screening tool and does not equate to clinical diagnosis. Cases identified through this instrument should undergo further in‐depth diagnostic assessments in subsequent studies.

In summary, circadian rhythm variations and dietary rhythmicity variations seem to be related to the risk of depression, anxiety, and stress among young adults. The results of our study suggest that reducing SJL and maintaining a regular diet could be potential measures to prevent symptoms of depression, anxiety, and stress, but the impact might be sex‐specific.

## Funding

This study was funded by the Hunan Provincial Natural Science Foundation of China (Grant 2025JJ50564, Yi‐de Yang), National Natural Science Foundation of China (Grant 81903336, Yi‐de Yang), National College Student Innovation and Entrepreneurship Training Program (Grant S202410542075, Zehui Fan), and Hunan Province Key Research and Development Project (Grant 2023SK2059, Xiu‐qin Hong).

## Disclosure

The funders had no role in the study design, data collection, data analysis, writing of this article, or interpretation of the results.

## Conflicts of Interest

The authors declare no conflicts of interest.

## Supporting Information

Additional supporting information can be found online in the Supporting Information section.

## Supporting information


**Supporting Information** Table S1: Classification criteria for DASS‐21 subscales. Table S2: Association between binary categorized behavior rhythmicity indicators and depression, anxiety and stress. Model 1: Crude model. Model 2: Adjusted for gender and age were adjusted. Model 3: Adjusted for gender, age, exercise time, screen time, being an only child, and family’s monthly per capita income were all adjusted. Table S3: Associations between behavior rhythmicity indicators with depression, anxiety, and stress scores based on DASS‐21. Table S4: Stratified analysis on the association between behavior rhythmicity indicators and risk of depression, anxiety and stress by gender. Table S5: Sensitivity analysis for the association between behavior rhythmicity indicators with risk of depression, anxiety, and stress defined by DASS‐21 and clinical diagnosis records. Figure S1: This schematic illustrates the calculations of social jetlag (SJL) and social jetlag sleep‐corrected (SJLsc).

## Data Availability

The data that support the findings of this study are available upon request from the corresponding author. The data are not publicly available due to privacy or ethical restrictions.
