# Supplementary material for: The Relationship Between Indicators of Behavior Rhythmicity With Depression, Anxiety, and Stress Among Youths
Source: Depress Anxiety. 2026 Aug 25;2026:5861864. doi: 10.1155/da/5861864 (PMC13504449; doi:10.1155/da/5861864)
Supplement: Supplementary file 1 [file DA-2026-5861864-s001.docx]

**Figure S1.** This schematic illustrates the calculations of social jetlag (SJL) and social jetlag sleep-corrected (SJL_sc_). SJL: social jetlag, SJL_sc_: social jetlag sleep-corrected, MSF: midsleep on free days, MSF_sc_=midsleep on free days sleep corrected, MSW: midsleep on workdays, MSW_sc_: midsleep on workdays sleep corrected, SD_w_=sleep duration on workdays, SD_f_ =sleep duration on work-free days, SD_week_=weekly average sleep duration, SO_f_=sleep onset on work-free days, SO_w_=sleep onset on work days.

**Table S1.** **Classification Criteria for DASS-21 Subscales**

|  | depression | anxiety | stress |
| --- | --- | --- | --- |
| normal | 0-9 | 0-7 | 0-14 |
| mild | 10-13 | 8-9 | 15-18 |
| moderate | 14-20 | 10-14 | 19-25 |
| severe | 21-27 | 15-19 | 26-33 |
| extremely severe | 28+ | 20+ | 34+ |

**Table S2. Associations between behavior rhythmicity indicators with risk of depression, anxiety and stress**

| Dependent variables | Independent variables |  | Model 1 | | Model 2 | | Model 3 | |
| --- | --- | --- | --- | --- | --- | --- | --- | --- |
|  |  |  | OR(95%CI) | *P* | OR(95%CI) | *P* | OR(95%CI) | *P* |
| depression | Social jet lag | <2h | 1(Ref.) |  | 1(Ref.) |  | 1(Ref.) |  |
|  |  | ≥2h | 1.463(0.473,4.522) | 0.509 | 1.529(0.491,4.759) | 0.464 | 1.439(0.459,4.512) | 0.533 |
|  | Sleep-corrected social jet lag | <2h | 1(Ref.) |  | 1(Ref.) |  | 1(Ref.) |  |
|  |  | ≥2h | 1.737(0.761,3.965) | 0.189 | 1.710(0.745,3.923) | 0.206 | 1.683(0.725,3.906) | 0.226 |
|  | First eating occasion jet lag | <2h | 1(Ref.) |  | 1(Ref.) |  | 1(Ref.) |  |
|  |  | ≥2h | 1.193(0.804,1.769) | 0.381 | 1.275(0.855,1.902) | 0.233 | 1.282(0.857,1.918) | 0.219 |
|  | Last eating occasion jet lag | <2h | 1(Ref.) |  | 1(Ref.) |  | 1(Ref.) |  |
|  |  | ≥2h | 1.378(0.787,2.412) | 0.262 | 1.309(0.744,2.302) | 0.350 | 1.356(0.767,2.395) | 0.294 |
|  | Eating window jet lag | <2h | 1(Ref.) |  | 1(Ref.) |  | 1(Ref.) |  |
|  |  | ≥2h | 1.294(0.870,1.922) | 0.203 | 1.359(0.911,2.028) | 0.133 | 1.423(0.949,2.134) | 0.088 |
| anxiety | Social jet lag | <2h | 1(Ref.) |  | 1(Ref.) |  | 1(Ref.) |  |
|  |  | ≥2h | 0.624(0.220,1.770) | 0.375 | 0.632(0.223,1.794) | 0.389 | 0.600(0.210,1.710) | 0.339 |
|  | Sleep-corrected social jet lag | <2h | 1(Ref.) |  | 1(Ref.) |  | 1(Ref.) |  |
|  |  | ≥2h | 1.915(0.941,3.899) | 0.073 | 1.921(0.942,3.916) | 0.072 | 1.884(0.918,3.867) | 0.084 |
|  | First eating occasion jet lag | <2h | 1(Ref.) |  | 1(Ref.) |  | 1(Ref.) |  |
|  |  | ≥2h | 1.213(0.898,1.638) | 0.207 | 1.218(0.900,1.649) | 0.201 | 1.213(0.895,1.645) | 0.213 |
|  | Last eating occasion jet lag | <2h | 1(Ref.) |  | 1(Ref.) |  | 1(Ref.) |  |
|  |  | ≥2h | 1.315(0.836,2.068) | 0.236 | 1.297(0.824,2.044) | 0.262 | 1.312(0.830,2.073) | 0.245 |
|  | Eating window jet lag | <2h | 1(Ref.) |  | 1(Ref.) |  | 1(Ref.) |  |
|  |  | ≥2h | 1.242(0.920,1.677) | 0.158 | 1.242(0.918,1.680) | 0.159 | 1.265(0.933,1.715) | 0.127 |
| stress | Social jet lag | <2h | 1(Ref.) |  | 1(Ref.) |  | 1(Ref.) |  |
|  |  | ≥2h | 0.354(0.047,2.690) | 0.316 | 0.355(0.047,2.700) | 0.317 | 0.340(0.044,2.599) | 0.298 |
|  | Sleep-corrected social jet lag | <2h | 1(Ref.) |  | 1(Ref.) |  | 1(Ref.) |  |
|  |  | ≥2h | 1.767(0.744,4.199) | 0.197 | 1.755(0.738,4.174) | 0.203 | 1.840(0.763,4.436) | 0.174 |
|  | First eating occasion jet lag | <2h | 1(Ref.) |  | 1(Ref.) |  | 1(Ref.) |  |
|  |  | ≥2h | 1.210(0.763,1.844) | 0.377 | 1.234(0.807,1.888) | 0.332 | 1.250(0.815,1.918) | 0.307 |
|  | Last eating occasion jet lag | <2h | 1(Ref.) |  | 1(Ref.) |  | 1(Ref.) |  |
|  |  | ≥2h | 1.873(1.072,3.272) | **0.028** | 1.865(1.065,3.265) | **0.029** | 1.964(1.113,3.466) | **0.020** |
|  | Eating window jet lag | <2h | 1(Ref.) |  | 1(Ref.) |  | 1(Ref.) |  |
|  |  | ≥2h | 1.328(0.869,2.030) | 0.191 | 1.349(0.887,2.066) | 0.169 | 1.439(0.935,2.217) | 0.098 |

Model1: crude model. Model 2: adjusted for gender and age were adjusted. Model 3: adjusted for gender, age, exercise time, screen time, being an only child, and family’s monthly per capita income were all adjusted.

**Table S3. Associations between behavior rhythmicity indicators with depression, anxiety and stress score based on DASS-21**

| Dependent variables | | Independent variables | Model 1 | | Model 2 | | Model 3 | |
| --- | --- | --- | --- | --- | --- | --- | --- | --- |
|  |  |  | β（95%CI） | *P* | β（95%CI） | *P* | β（95%CI） | *P* |
| Depression | Eating window | | -0.136(-0.386,0.113) | 0.284 | -0.163(-0.412,0.087) | 0.200 | -0.16(-0.409,0.089) | 0.207 |
|  | Social jet lag | | 0.872(-0.105,1.849) | 0.080 | 1.011(0.035,1.987) | **0.042** | 0.963(-0.011-1.936) | 0.053 |
|  | Sleep-corrected social jet lag | | 1.799(0.922,2.675) | **＜0.001** | 1.790(0.918,2.663) | **＜0.001** | 1.715(0.844,2.586) | **＜0.001** |
|  | First eating occasion jet lag | | 0.267(-0.045,0.580) | 0.093 | 0.337(0.024,0.650) | **0.035** | 0.321(0.009,0.633) | **0.044** |
|  | Last eating occasion jet lag | | 0.422(-0.042,0.886) | 0.075 | 0.392(-0.071,0.855) | 0.097 | 0.411(-0.050,0.872) | 0.080 |
|  | Eating window jet lag | | 0.116(-0.179,0.412) | 0.44 | 0.151(-0.143,0.446) | 0.314 | 0.160(-0.134,0.454) | 0.285 |
|  | Sleep duration | | -0.600(-1.379,0.180) | 0.131 | -0.506(-1.285,0.272) | 0.202 | -0.432(-1.211,0.347) | 0.276 |
|  | Chronotype | | 2.049(0.927,3.170) | **＜0.001** | 2.293(1.171,3.414) | **＜0.001** | 2.144(1.018,3.269) | **＜0.001** |
|  | breakfast skipping | | 0.978(-0.145,2.101) | 0.088 | 1.003(-0.116,2.122) | 0.079 | 0.874(-0.245,1.994) | 0.126 |
| Anxiety | Eating window | | 0.089(-0.141,0.319) | 0.450 | 0.087(-0.144,0.319) | 0.458 | 0.09(-0.141,0.321) | 0.444 |
|  | Social jet lag | | 0.170(-0.732,1.072) | 0.712 | 0.137(-0.770,1.044) | 0.766 | 0.102(-0.803,1.007) | 0.825 |
|  | Sleep-corrected social jet lag | | 1.448(0.640,2.256) | **＜0.001** | 1.454(0.646,2.263) | **＜0.001** | 1.392(0.584,2.199) | **＜0.001** |
|  | First eating occasion jet lag | | 0.439(0.152,0.726) | **0.003** | 0.438(0.148,0.727) | **0.003** | 0.427(0.139,0.716) | **0.004** |
|  | Last eating occasion jet lag | | 0.503(0.076,0.930) | **0.021** | 0.502(0.074,0.930) | **0.022** | 0.522(0.095,0.948) | **0.017** |
|  | Eating window jet lag | | 0.297(0.025,0.568) | **0.032** | 0.291(0.018,0.563) | **0.037** | 0.307(0.035,0.579) | **0.027** |
|  | Sleep duration | | -0.631(-1.349,0.086) | 0.085 | -0.64(-1.361,0.082) | 0.082 | -0.561(-1.282,0.161) | 0.128 |
|  | Chronotype | | 2.216(1.186,3.246) | **＜0.001** | 2.234(1.195,3.273) | **＜0.001** | 2.094(1.052,3.136) | **＜0.001** |
|  | breakfast skipping | | 0.481(-0.556,1.517) | 0.363 | 0.501(-0.538,1.541) | 0.947 | 0.371(-0.668,1.410) | 0.483 |
| Stress | Eating window | | 0.038(-0.244,0.320) | 0.792 | 0.050(-0.234,0.333) | 0.731 | 0.050(-0.233,0.333) | 0.729 |
|  | Social jet lag | | -0.188(-1.294,0.918) | 0.739 | -0.129(-1.239,0.981) | 0.820 | -0.161(-1.269,0.947) | 0.776 |
|  | Sleep-corrected social jet lag | | 1.659(0.666,2.652) | **0.001** | 1.644(0.651,2.637) | **0.001** | 1.577(0.585,2.569) | **0.002** |
|  | First eating occasion jet lag | | 0.197(-0.156,0.550) | 0.273 | 0.209(-0.147,0.565) | 0.250 | 0.198(-0.157,0.553) | 0.274 |
|  | Last eating occasion jet lag | | 0.927(0.406,1.448) | **＜0.001** | 0.950(0.429,1.472) | **＜0.001** | 0.974(0.454,1.494) | **＜0.001** |
|  | Eating window jet lag | | 0.251(-0.083,0.584) | 0.140 | 0.265(,0.069,0.599) | 0.120 | 0.287(-0.047,0.620) | 0.092 |
|  | Sleep duration | | -0.556(-1.437,0.324) | 0.215 | -0.579(-1.463,0.305) | 0.199 | -0.478(-1.362,0.406) | 0.288 |
|  | chronotype | | 1.882(0.612,3.152) | **0.004** | 1.906(0.626,3.186) | **0.004** | 1.742(0.458,3.026) | **0.008** |
|  | breakfast skipping | | 0.125(-1.146,1.396) | 0.847 | 0.070(-1.203,1.343) | 0.914 | -0.080(-1.353,1.192) | 0.901 |
| Total | Eating window | | -0.010(-0.688,0.669) | 0.977 | -0.026(-0.707,0.656) | 0.941 | -0.020(-0.700,0.660) | 0.954 |
|  | Social jet lag | | 0.853(-1.805,3.512) | 0.529 | 1.019(-1.652,3.691) | 0.454 | 0.904(-1.757,3.565) | 0.505 |
|  | Sleep-corrected social jet lag | | 4.906(2.527,7.285) | **＜0.001** | 4.889(2.509,7.269) | **＜0.001** | 4.684(2.310,7.057) | **＜0.001** |
|  | First eating occasion jet lag | | 0.904(0.056,1.751) | **0.037** | 0.984(0.129,1.839) | **0.024** | 0.946(0.095,1.797) | **0.029** |
|  | Last eating occasion jet lag | | 1.852(0.596,3.108) | **0.004** | 1.844(0.585,3.104) | **0.004** | 1.907(0.654,3.159) | **0.003** |
|  | Eating window jet lag | | 0.664(-0.138,1.465) | 0.104 | 0.707(-0.097,1.511) | 0.085 | 0.754(-0.047,1.555) | 0.065 |
|  | Sleep duration | | -1.787(-3.903,0.328) | 0.098 | -1.725(-3.851,0.401) | 0.112 | -1.471(-3.594,0.652) | 0.174 |
|  | chronotype | | 6.146(3.107,9.186) | **＜0.001** | 6.433(3.371,9.495) | **＜0.001** | 5.980(2.911,9.048) | **＜0.001** |
|  | breakfast skipping | | 1.584(-1.471,4.639) | 0.309 | 1.574(-1.487,4.636) | 0.313 | 1.165(-1.892,4.222) | 0.454 |

**Table S4. Stratified analysis on the association between behavior rhythmicity indicators and risk of depression, anxiety and stress by gender**

| Variables |  | Male | | | Female | | | *P_interaction_* |
| --- | --- | --- | --- | --- | --- | --- | --- | --- |
|  |  | *OR* | *95%CI* | *P* | *OR* | *95%CI* | *P* |  |
| Depression |  |  |  |  |  |  |  |  |
| Sleep duration |  | 0.867 | 0.512~1.468 | 0.595 | 0.908 | 0.638~1.292 | 0.592 | 0.991 |
| Eating window |  | 0.998 | 0.862~1.155 | 0.976 | 0.909 | 0.807~1.024 | 0.115 | 0.921 |
| Social jet lag |  | 1.541 | 0.878~2.705 | 0.132 | 1.306 | 0.839~2.033 | 0.237 | 0.745 |
| Sleep-corrected social jet lag |  | 2.014 | 1.213~3.345 | **0.007** | 1.269 | 0.831~1.940 | 0.27 | 0.216 |
| First eating occasion jet lag |  | 1.066 | 0.874~1.300 | 0.529 | 1.131 | 0.982~1.303 | 0.088 | 0.552 |
| Last eating occasion jet lag |  | 1.112 | 0.857~1.443 | 0.423 | 1.087 | 0.885~1.336 | 0.426 | 0.907 |
| Eating window jet lag |  | 0.978 | 0.802~1.193 | 0.825 | 1.076 | 0.945~1.224 | 0.269 | 0.421 |
| chronotype | non-evening type(Ref.) |  | 1 |  |  | 1 |  |  |
|  | evening type | 1.770 | 0.835~3.750 | 0.136 | 1.853 | 1.130~3.037 | **0.014** | 0.842 |
| breakfast skipping | 7day/week (Ref.) |  | 1 |  |  | 1 |  |  |
|  | <7day/week | 1.658 | 0.797~3.448 | 0.176 | 1.113 | 0.658~1.883 | 0.689 | 0.451 |
| Sleep duration | ≥7h(Ref.) |  | 1 |  |  | 1 |  |  |
|  | <7h | 1.431 | 0.676~3.029 | 0.349 | 1.364 | 0.796~2.338 | 0.259 | 0.973 |
| Anxiety |  |  |  |  |  |  |  |  |
| Sleep duration |  | 0.875 | 0.558~1.373 | 0.562 | 0.894 | 0.692~1.156 | 0.393 | 0.898 |
| Eating window |  | 1.034 | 0.912~1.173 | 0.601 | 0.979 | 0.898~1.067 | 0.628 | 0.471 |
| Social jet lag |  | 1.411 | 0.855~2.328 | 0.178 | 0.823 | 0.588~1.152 | 0.257 | 0.079 |
| Sleep-corrected social jet lag |  | 1.524 | 0.980~2.371 | 0.062 | 1.216 | 0.884~1.672 | 0.23 | 0.421 |
| First eating occasion jet lag |  | 1.100 | 0.927~1.305 | 0.276 | 1.06 | 0.955~1.176 | 0.276 | 0.692 |
| Last eating occasion jet lag |  | 1.166 | 0.928~1.465 | 0.187 | 1.109 | 0.948~1.298 | 0.196 | 0.775 |
| Eating window jet lag |  | 1.114 | 0.944~1.315 | 0.202 | 1.058 | 0.961~1.166 | 0.250 | 0.960 |
| chronotype | non-evening type(Ref.) |  | 1 |  |  | 1 |  |  |
|  | evening type | 2.013 | 1.031~3.929 | **0.040** | 1.453 | 1.009~2.093 | **0.044** | 0.374 |
| breakfast skipping | 7day/week (Ref.) |  | 1 |  |  | 1 |  |  |
|  | <7day/week | 1.213 | 0.660~2.227 | 0.534 | 1.037 | 0.714~1.507 | 0.848 | 0.658 |
| Sleep duration | ≥7h(Ref.) |  | 1 |  |  | 1 |  |  |
|  | <7h | 1.207 | 0.627~2.324 | 0.574 | 1.291 | 0.861~1.934 | 0.216 | 0.911 |
| Stress |  |  |  |  |  |  |  |  |
| Sleep duration |  | 0.737 | 0.396~1.370 | 0.335 | 0.882 | 0.612~1.272 | 0.501 | 0.581 |
| Eating window |  | 0.914 | 0.767~1.090 | 0.318 | 1.079 | 0.954~1.220 | 0.228 | 0.130 |
| Social jet lag |  | 1.247 | 0.661~2.351 | 0.495 | 0.787 | 0.481~1.286 | 0.339 | 0.250 |
| Sleep-corrected social jet lag |  | 2.066 | 1.168~3.654 | **0.013** | 1.246 | 0.805~1.928 | 0.324 | 0.165 |
| First eating occasion jet lag |  | 0.995 | 0.790~1.253 | 0.965 | 1.078 | 0.931~1.247 | 0.316 | 0.543 |
| Last eating occasion jet lag |  | 1.383 | 1.035~1.846 | **0.028** | 1.16 | 0.950~1.416 | 0.145 | 0.456 |
| Eating window jet lag |  | 0.965 | 0.763~1.220 | 0.765 | 1.155 | 1.015~1.315 | **0.029** | 0.106 |
| chronotype | non-evening type(Ref.) |  | 1 |  |  | 1 |  |  |
|  | evening type | 1.624 | 0.690~3.820 | 0.267 | 2.031 | 1.227~3.363 | **0.006** | 0.816 |
| breakfast skipping | <7day/week |  | 1 |  |  | 1 |  |  |
|  | ≥7h(Ref.) | 0.818 | 0.354~1.889 | 0.637 | 0.941 | 0.556~1.591 | 0.820 | 0.920 |
| Sleep duration | ≥7h(Ref.) |  | 1 |  |  | 1 |  |  |
|  | <7h | 1.634 | 0.689~3.876 | 0.266 | 1.023 | 0.575~1.823 | 0.937 | 0.358 |

Gender, age, exercise time, screen time, being an only child, and family’s monthly per capita income were all adjusted.

**Table S5. Sensitivity analysis for the association between behavior rhythmicity indicators with risk of depression, anxiety and stress defined by DASS-21 and clinical diagnosis records**

| Dependent variables | | Independent variables | p | Model 1 | | | Model 2 | | | | Model 3 | | |
| --- | --- | --- | --- | --- | --- | --- | --- | --- | --- | --- | --- | --- | --- |
|  |  |  |  | OR (95%CI) | *P* | | OR (95%CI) | | *P* | | OR (95%CI) | |  |
| Outcome 1* | Eating window | |  | 1.033(0.922-1.156) | 0.577 | | 1.022(0.912-1.145) | | 0.705 | | 1.015(0.903-1.141) | | 0.805 |
|  | Social jet lag | |  | 1.195(0.794-1.799) | 0.392 | | 1.185(0.785-1.788) | | 0.419 | | 1.138(0.749-1.728) | | 0.545 |
|  | Sleep-corrected social jet lag | |  | 1.725(1.177-2.530) | **0.005** | | 1.721(1.172-2.528) | | **0.006** | | 1.788(1.208-2.647) | | **0.004** |
|  | First eating occasion jet lag | |  | 1.110(0.969-1.271) | 0.132 | | 1.109(0.967-1.273) | | 0.140 | | 1.100(0.955-1.267) | | 0.185 |
|  | Last eating occasion jet lag | |  | 1.218(1.023-1.452) | **0.027** | | 1.212(1.016-1.446) | | **0.032** | | 1.214(1.014-1.453) | | **0.035** |
|  | Eating window jet lag | |  | 1.193(1.048-1.358) | **0.007** | | 1.186(1.041-1.352) | | **0.011** | | 1.185(1.037-1.354) | | **0.013** |
|  | Sleep duration | | >7h | 1(Ref.) |  | | 1(Ref.) | |  | | 1(Ref.) | |  |
|  |  | | ≤7h | 1.437(0.848-2.437) | 0.178 | | 1.399(0.823-2.378) | | 0.215 | | 1.383(0.806-2.375) | | 0.239 |
|  | Chronotype | | non-evening type | 1(Ref.) |  | | 1(Ref.) | |  | | 1(Ref.) | |  |
|  |  | | evening type | 2.036(1.245-3.329) | **0.005** | | 2.081(1.263-3.429) | | **0.004** | | 2.089(1.249-3.495) | | **0.005** |
|  | Chronotype | | Definitely Morning Type | NA |  | NA | |  | | NA | |  | |
|  |  | | Moderately Morning Type | 1(Ref.) |  | 1(Ref.) | |  | | 1(Ref.) | |  | |
|  |  | | Neither Type | 1.482(0.323-6.804) | 0.613 | 1.442(0.313-6.649) | | 0.639 | | 0.782(0.284-2.154) | | 0.671 | |
|  |  | | Moderately Evening Type | 2.889(0.620-13.470) | 0.177 | 2.865(0.612-13.403) | | 0.181 | | 1.524(0.544-4.270) | | 0.194 | |
|  |  | | Definitely Evening Type | 3.714(0.539-25.593) | 0.183 | 3.908(0.562-27.162) | | 0.168 | | 0.975(0.198-4.624) | | 0.259 | |
|  | breakfast skipping | | 7day/week | 1(Ref.) |  | | 1(Ref.) | |  | | 1(Ref.) | |  |
|  |  | | <7day/week | 0.909(0.546-1.515) | 0.715 | | 0.928(0.556-1.549) | | 0.775 | | 0.820(0.946-2.292) | | 0.463 |
| Outcome 2# | Eating window | |  | 1.030(0.948-1.120) | 0.482 | | 1.024(0.942-1.114) | | 0.574 | | 1.025(0.941-1.116) | | 0.567 |
|  | Social jet lag | |  | 1.016(0.741-1.392) | 0.924 | | 1.010(0.736-1.387) | | 0.952 | | 0.988(0.717-1.360) | | 0.941 |
|  | Sleep-corrected social jet lag | |  | 1.372(1.007-1.868) | **0.045** | | 1.375(1.009-1.873) | | **0.044** | | 1.403(1.025-1.919) | | **0.034** |
|  | First eating occasion jet lag | |  | 1.068(0.963-1.185) | 0.212 | | 1.072(0.965-1.191) | | 0.197 | | 1.065(0.958-1.184) | | 0.245 |
|  | Last eating occasion jet lag | |  | 1.075(0.925-1.248) | 0.346 | | 1.068(0.919-1.241) | | 0.389 | | 1.071(0.920-1.245) | | 0.377 |
|  | Eating window jet lag | |  | 1.077(0.977-1.188) | 0.135 | | 1.075(0.975-1.187) | | 0.148 | | 1.071(0.970-1.183) | | 0.174 |
|  | Sleep duration | | >7h | 1(Ref.) |  | | 1(Ref.) | |  | | 1(Ref.) | |  |
|  |  | | ≤7h | 1.160(0.773-1.741) | 0.474 | | 1.134(0.754-1.706) | | 0.545 | | 1.118(0.740-1.688) | | 0.596 |
|  | Chronotype | | non-evening type | 1(Ref.) |  | | 1(Ref.) | |  | | 1(Ref.) | |  |
|  |  | | evening type | 1.502(1.035-2.180) | **0.032** | | 1.542(1.057-2.249) | | **0.025** | | 1.538(1.049-2.256) | | **0.028** |
|  | Chronotype | | Definitely Morning Type | NA |  | | NA | |  | | NA | |  |
|  |  | | Moderately Morning Type | 1(Ref.) |  | | 1(Ref.) | |  | | 1(Ref.) | |  |
|  |  | | Neither Type | 1.635(0.569-4.700) | 0.361 | | 1.594(0.553-4.594) | | 0.388 | | 1.524(0.522-4.452) | | 0.441 |
|  |  | | Moderately Evening Type | 2.378(0.809-6.988) | 0.115 | | 2.375(0.806-7.002) | | 0.117 | | 2.283(0.764-6.826) | | 0.140 |
|  |  | | Definitely Evening Type | 2.600(0.598-11.310) | 0.203 | | 2.706(0.619-11.841) | | 0.186 | | 2.430(0.547-10.793) | | 0.243 |
|  | breakfast skipping | | 7day/week | 1(Ref.) |  | | 1(Ref.) | |  | | 1(Ref.) | |  |
|  |  | | <7day/week | 0.766(0.528-1.110) | 0.159 | | 0.774(0.533-1.123) | | 0.177 | | 0.728(0.498-1.063) | | 0.101 |

*For the Outcome 1: cases included participants with positive clinical diagnosis or/and severe or extremely severe symptoms on the DASS-21 scale; controls consisted of participants with negative clinical diagnoses and DASS-21 scores ranging from normal to mild symptom severity.

#For the Outcome 2: cases covered individuals with positive clinical diagnosis or/and moderate, severe or extremely severe DASS-21 scores; controls consisted of participants with negative clinical diagnoses and DASS-21 scores ranging from normal to mild symptom severity.
